# Inherited and De Novo Variation in Lithuanian Genomes: Introduction to the Analysis of the Generational Shift

**DOI:** 10.3390/genes13040569

**Published:** 2022-03-23

**Authors:** Alina Urnikyte, Laura Pranckeniene, Ingrida Domarkiene, Svetlana Dauengauer-Kirliene, Alma Molyte, Ausra Matuleviciene, Ingrida Pilypiene, Vaidutis Kučinskas

**Affiliations:** Department of Human and Medical Genetics, Biomedical Science Institute, Faculty of Medicine, Vilnius University, Santariskiu Street 2, LT-08661 Vilnius, Lithuania; ingrida.domarkiene@mf.vu.lt (I.D.); svetlana.dauengauer-kirliene@mf.vu.lt (S.D.-K.); alma.molyte@mf.vu.lt (A.M.); ausra.matuleviciene@mf.vu.lt (A.M.); ingrida.pilypiene@mf.vu.lt (I.P.); vaidutis.kucinskas@mf.vu.lt (V.K.)

**Keywords:** whole genome sequencing, SNV, de novo variation, newborns, trios

## Abstract

Most genetic variants are rare and specific to the population, highlighting the importance of characterizing local population genetic diversity. Many countries have initiated population-based whole-genome sequencing (WGS) studies. Genomic variation within Lithuanian families are not available in the public databases. Here, we describe initial findings of a high-coverage (an average of 36.27×) whole genome sequencing for 25 trios of the Lithuanian population. Each genome on average carried approximately 4,701,473 (±28,255) variants, where 80.6% (3,787,626) were single nucleotide polymorphisms (SNPs), and the rest 19.4% were indels. An average of 12.45% was novel according to dbSNP (build 150). The WGS structural variation (SV) analysis identified on average 9133 (±85.10) SVs, of which 95.85% were novel. De novo single nucleotide variation (SNV) analysis identified 4417 variants, where 1.1% de novo SNVs were exonic, 43.9% intronic, 51.9% intergenic, and the rest 3.13% in UTR or downstream sequence. Three potential pathogenic de novo variants in the *ZSWIM8*, *CDC42EP1*, and *RELA* genes were identified. Our findings provide useful information on local human population genomic variation, especially for de novo variants, and will be a valuable resource for further genetic studies, and medical implications.

## 1. Introduction

In recent years, the accessibility of whole-genome sequencing (WGS) together with new computational and statistical methods has allowed us to not only to analyze genomic patterns of variation, but also infer de novo mutations and selection events, and unravel their potential phenotypic consequences under different environmental conditions. To identify which region of the human genome might be evolving, we first need to identify mutation prevalence changes across different generations and test their effect on fitness. 

Lithuania is a country in the Baltic region of Europe with a population of 2.8 million. Previous research has revealed the Lithuanian population’s partial isolation and genetic distinctiveness within the European context [1]. Structure analysis performed by Urnikyte et al., 2021 [2] identified the close genetic proximity of Lithuanians to Latvians, Estonians and Belarusians, with moderate impact of Finno-Ugrians on Lithuanians. Many articles about various inherited or single de novo mutations identified in the Lithuanian families related to specific pathologies can be found in the NCBI database. However, there is no summarizing data that could cover whole-genome sequencing data for the Lithuanian families nor for inherited, nor for the novel genome variants for at least one generation. Summarized de novo germline variants were studied only in one whole exome general Lithuanian population scale study. According to previous (Pranckeniene et al., 2018) research, the rate of de novo variants (DNV) was identified as significantly higher than in other population studies—2.4 × 10^−8^ and 2.74 × 10^−8^ for single nucleotides, and for de novo indels it was 1.77 × 10^−8^ per position per generation [3]. The higher DNV rate was elucidated by the only exome analysis model whereas exomes exhibit significantly higher (by 30%) mutation rates than whole genomes regarding the base pair composition of the whole genome is different from that of exomes. Researches showed that DNAse 1 hypersensitivity, context of CpG islands, GERPP++ conservation values, and expression level explained 68–93% of the DNV rate. There also four possible pathogenic DNVs were found in the genes encoding proteins that are essential for chromatin modeling, regulation of the cytoskeleton, modulation of cell growth and vitality, function of cytoplasmic signaling pathways, and initiation of neuronal response. These variants were not deleterious enough to reduce mean fitness, therefore individuals with these novel variants are healthy. 

Many countries have initiated population-based WGS studies [4,5,6]. However, there are still many geographical regions, for example, Lithuania, lacking genomic information in public-databases. Previous population studies have been performed using exome sequencing [3,7] or genome-wide genotyping [1,8,9] data. To widen our knowledge of patterns of genomic variation in the Lithuanian population, we aimed to analyze high-coverage (an average of 36.27×, see Table S1) genome sequencing data of 25 trios. This study represents the first WGS data analysis of the genome variation within Lithuanian families providing more comprehensive characterization of local human population genomic variation, especially for de novo, and structural variants.

## 2. Materials and Methods

### 2.1. Study Population

In this study, Lithuanian families who lived in Lithuania for at least three generations according to the pedigree were included. A total of 35 trios were collected to the study group: 35 newborns and 70 parents (105 individuals in total). Two standardized questionnaires about participants’ behavior and medical data were filled by investigators. Only healthy Lithuanian nationality adult participants were included in the cohort. The criteria for the inclusion of newborns were: born to Lithuanian parents, a healthy term-born neonate with a population mean birth weight. Twenty-five trios meeting the established inclusion criteria were selected for the whole-genome sequencing. After digitizing the collected questionnaires and performing the analysis of descriptive statistics, we found that maternal and paternal age was 30.4 (±3.62) and 33.96 (±4.88) years old on average, respectively. All the newborns were screened for whole blood count and leucogram, C reactive protein, pH from the umbilical cord, blood group, Rh factor as well as for inherited abnormalities using head, heart, abdominal and renal ultrasound. Based on the total laboratory and instrumental data, only those who had no pathologies were considered healthy. All 25 newborns enrolled in the study (17 (62%) boys and 8 (32%) girls) were born naturally at a median gestational age of 39.70 (±0.93) weeks in 96% from the primiparous pregnancy. The weight, height, and head circumference of all neonates were in line with the Lithuanian population average regardless of gender. The mean neonatal weight was 3580.8 g (±326.48 g), height was 54.04 cm (±1.65 cm), and head circumference was 35.64 cm ± 0.86 cm.

Demographic and health information of the newborn’s parents, data on the pregnancy and childbirth history, and data from a newborn clinical examination and laboratory and instrumental studies were collected. DNA samples from parental venous blood and neonatal umbilical cord blood were collected for whole-genome sequencing. DNA was extracted from whole blood (3 mL) using QIAGEN GENTRA^®^ Puregene^®^ Blood Kit (Qiagen GmbH) according to the manufacturer’s protocol. DNA concentration and quality were assessed using NanoDropR ND-1000 spectrophotometer (NanoDrop Technologies Inc., Wilmington, DE, USA).

### 2.2. DNA Sequencing

Whole-genome sequencing (WGS) was performed for 25 trios of Lithuanian origin at coverage of 26.88–61.38× (an average of 36.27×), Table S1. Overall, the sample size was 75 individuals: 25 newborns, 25 mothers, and 25 fathers. WGS was performed at the CeGaT company (Tubingen, Germany). 100 ng DNA was paired-end sequenced in 2 × 150 bp mode on state-of-the-art Illumina NovaSeq™ 6000 Sequencing System using TruSeq^®^ Nano DNA Library Prep Kit (Illumina Inc., San Diego, CA, USA). 

Demultiplexing of the sequencing reads was performed with Illumina bcl2fastq (2.20). Adapters were trimmed with Skewer (version 0.2.2) [10]. Quality trimming of the reads has not been performed. Analysis of sequencing data was performed using the Illumina DRAGEN platform (version 3.6.4). The DRAGEN DNA Pipeline uses the current industry standard, BWA-MEM and GATK-HC software. Reads were mapped to the reference genome hg19 (present on the Illumina DRAGEN platform v.3.6.4) and duplicates were marked. Calling of small variants, regions of homozygosity, and structural variants was performed with default parameters. SNVs found at higher frequencies than 1% in the population were qualified as SNPs. The quality of the FASTQ files was analyzed with FastQC (version 0.11.5-cegat) [11]. Sequencing quality control Q30 values were above 88.59% (Figures S1–S3).

### 2.3. Structural Variation Detection

Calling of structural variants (SV) such as translocations, inversions, large and medium-sized indels was performed by Illumina DRAGEN platform v.3.6.4 using the same methods as Manta [12]. Two individuals were removed from further analysis as the outliers according to the number of SVs. We have calculated mean, median, mode for deletions, duplications, and insertions in parent and newborn groups. The comparison between groups was performed using R v.4.0.2. [13], with a significance level of 0.05. To determine known and new SVs, SV annotation was performed on *.vcf* format files using AnnotSV software [13] with default parameters. After annotation, we analyzed only SVs with the FILTER status: PASS. Novel SVs were determined according to the annotation from gnomAD [14], ClinVar [15], ClinGen [16], DGV (dgv, nsv or esv) [17], DDD [18], 1000 genomes [19], Ira M. Hall’s lab [20], and Children’s Mercy Research Institute data [21].

### 2.4. De Novo Mutation Detection

To detect de novo variants, the whole genomes in *.bam* format of each family that consisted of father, mother, and child were combined using Samtools [22]. Initial identification of de novo variants was performed using the merged trio’s *.bam* file and the open-access VarScan v.2.4.4 software [23]. A potential de novo variant is identified if the child has a genomic variant when neither parent has it in the same genome position. The results are provided in a generated *.vcf* format data file. SnpSift v.4 software [24] was used for the initial rejection of false-positive results derived from VarScan [23]. The following conservative filtering criteria were applied: (1) a genotype quality of the individual ≥ 50; and (2) the number of reads at each site > 30.

Furthermore, to discard the remaining variants that were somatic (only present in a fraction of the sequenced blood cells) with low allele balance or sequencing artefacts, de novo variants were filtered by setting a threshold for the observed fraction of the reads in individuals with the alternative allele (the allele balance) for the trios [0.3; 0.7]. In cases where de novo variants were detected significantly more frequently than in all other trios, biological paternity verification using WGS data of specific regions was performed.

### 2.5. Variant Analysis

Prior to the analysis we carried out principal component analysis (PCA) to both sample groups: parents and newborns. PCA was performed with SmartPCA from EIGENSOFT 7.2.1 [25] using independent SNPs obtained with the indep-pairwise option of PLINK v.1.07 [26] with parameters: window size of 50 SNPs, a step size of 5, and a r^2^ threshold of 0.5. Genetic relationship was computed with VCFtools (0.1.16) [27] option relatedness. Samples detected as outliers in PCA plot (Figures S4 and S5) as well as individuals with relatedness coefficient higher than expected for unrelated individuals were removed from further analysis (Tables S5 and S6). To perform the analysis of the variants allele frequency distribution, all identified SNPs were annotated using gnomAD genome database (v.2.1.1) [14] in ANNOVAR [23] software. SNP frequencies determined by the gnomAD genome data were compared with the frequencies of the same SNPs in the Lithuanian population. Data visualization was performed by R software Rcmdr package (version 2.7-2) [28]. The structure analysis was carried out on the merged 1000 Genomes Project Phase3 [19] dataset with the SmartPCA program from EIGENSOFT 7.2.1 [25].

### 2.6. Variant Annotation

Single nucleotide variant annotation was performed using ANNOVAR v.20210123 [23] using hg19, cytoBand [29], RefSeqGene [30], avsnp150, dbnsfp30a [31], dbnsfp31a_interpro [31], dbnsfp33a [31], exac03 [32], kaviar_20150923 [33] SIFT [34], PolyPhen [32], LRT, MutationTaster [35], MutationAssessor [36], FATHMM [37], PROVEAN [38], CADD [39], GERP++ [40], PhyloP [41], SiPhy [42], and COSMIC [41] algorithm annotation tools. Databases providing information on the gene identifying the de novo variant and the dbSNP database [43] were also added, thus assigning the *rs* code to each genome variant, evaluating the genomic SNVs association with pathogenicity in ClinVar [15], evaluating the 1000 Genome Project Phase 3 dataset [19], and ExAC database [44] genome variant frequencies in different populations. For the de novo variants annotation Gene4Denovo201907 database [45] was used additionally. 

## 3. Results

### 3.1. Sample Collection

We found that 25 women included in the statistical analysis were 30.4 (±3.62) years of age on average, first-time delivering with higher education, did not smoke, and did not use drugs. Most of them (88%) did not complain about their health condition, a third (36%) did not drink alcohol at all. Overall, 64% of respondents reported consuming alcohol before pregnancy. Additionally, 88% of women took dietary supplements during pregnancy, and 96% of them used folic acid. Prenatal screening for chromosomal abnormalities was performed in 29% of cases. The risk of infection was minimal for most of the pregnant women because the amounts of amniotic fluid were within the normal range in 92%, 80% of group B streptococcal tests were negative, nobody had asymptomatic bacteriuria, and a maximum latency period did not exceed 18 hours. The mean age of the 25 fathers was 33.96 (±4.88) years. Overall, 84% of them had higher education. Most of them (88%) reported consuming alcohol in moderation and 16% smoking. The most detected ABO blood groups were B (44%) and O (32%), and the Rh factor was positive in 72% of cases. An increase in C-reactive protein on the first day after birth was observed in 20.83% of cases, therefore, seven infants underwent recurrent inflammatory markers: CRB and leukogram counts.

### 3.2. Genomic Variation Characterization 

A total of 25 trios of Lithuanian origin were sequenced at coverage of 26.88–61.38× (an average of 36.27×). On average, 94.72% of the reads were mapped to the reference genome hg19. Statistics of mapped reads are shown in Table S1. Sequencing quality control Q30 values were above 88.59% (Figures S1–S3). As presented in Table 1, after variant calling, an average of 4,704,096 SNVs per genome were discovered across autosomes in the parent group and 4,696,226 in the newborn cohort, with a transition/transversion ratio of 2.03, and the heterozygote/homozygote (het/hom) ratio of 1.6 (Tables S2–S4). In total, an average of 446,107 insertions and 433,072 deletions in parents, and 430,017 insertions and 442,233 deletions in newborns were identified (Tables S1–S3). The estimated het/hom ratios were 1.67−1.87 for insertions and 1.74−1.97 for deletions in both groups.

On average, we identified 3,791,674 SNPs per genome in parents and 3,783,584 SNPs in newborns (Table 1). Of all newborn chromosomes 88.8% SNPs were present in dbSNP (build 150) and 11.2% were novel, and in the parent group 13.7% were novel according to dbSNP (build 150) [43].

We detected on average 120,300 (2.5%) SNPs and indels in X and Y chromosomes. Based on variant distribution across different genomic regions 0.9% SNPs and <49 bp indels are located in exonic regions, 41.5% in intronic, 54.1% in intergenic, and 3.6% in downstream, and upstream sequences of the genome. The distribution of SNVs in genome sections is different: 0.24% is in the exonic regions, 24.5% in the intronic, 30.9% in the intergenic, and 44.4% in the downstream, upstream sequences of the genome (Figures S6 and S7). Each variant was annotated using ANNOVAR software [23] (see Section 2). 

**Figure 1:**
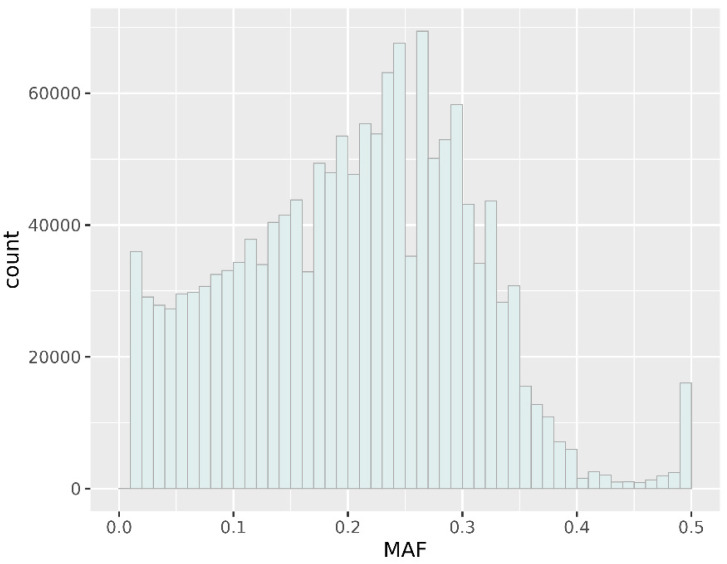
The minor allele frequency distribution for 1,508,407 SNPs in the Lithuanian population samples.

The analysis of the variant allele frequency collected in the gnomAD [14] database, compared to the distribution of the same genome variants in Lithuanian genomes, showed that the Lithuanian population is distinguished by alleles whose frequency in the gnomAd database is identified as rare or unique. This is best reflected by comparing allele frequencies between the Lithuanian and African American, Finish, uncertain and Ashkenazi Jewish ancestry genomes (Figure S8). An exclusive comparison of results of the Lithuanian genome variants with Ashkenazi Jewish (AJN) ancestry genomes indicates that about half of the common variants with frequency 0.05–0.5 present in AJN genomes, in Lithuanian’s genomes becomes rare allelic variants with minor allele frequency (MAF) < 0.05. Assessing the distribution of MAF for the Lithuanian population data set of 1,508,407 SNPs identified 4.3% SNVs are rare with MAF ≤ 0.01, and 9.5% are low frequency (MAF 0.01–0.05), and the rest are common (MAF > 0.05) (Figure 1). 

To infer population structure we combined Lithuanian and 1000 Genomes Project Phase3 [19] dataset, generating a pooled dataset of 242,188 autosomal SNPs in a total of 2553 individuals. We analyzed 28 populations from main geographical regions: Africa including the Yoruba in Ibadan, Nigeria (YRI), Luhya in Webuye, Kenya (LWK), Gambian in Western Divisions in the Gambia (GWD), Mende in Sierra Leone (MSL), and Esan in Nigeria (ESN) populations; Europe including Utah residents with ancestry from northern and western Europe (CEU), Toscani in Italy (TSI), Finnish in Finland (FIN), British in England and Scotland (GBR), Lithuanians (LT), Iberian population from Spain (IBS); East Asia including Han Chinese in Bejing, China (CHB), Japanese in Tokyo, Japan (JPT), Southern Han Chinese, China (CHS), Chinese Dai in Xishuangbanna, China (CDX), Kinh in Ho Chi Minh City, Vietnam (KHV), Denver Chinese in Denver, Colorado (CHD); South Asia including Gujarati Indians in Houston, Texas (GIH), Punjabi from Lahore, Pakistan (PJL), Bengali from Bangladesh (BEB), Sri Lankan Tamil from the UK (STU) and Indian Telugu from the UK (ITU); America including African American in Southwest US (ASW), African Caribbean in Barbados (ACB), Mexican-American in Los Angeles, California (MXL), Puerto Rican in Puerto Rico (PUR), Colombian in Medellin, Colombia (CLM), and Peruvian in Lima, Peru (PEL).

**Figure 2:**
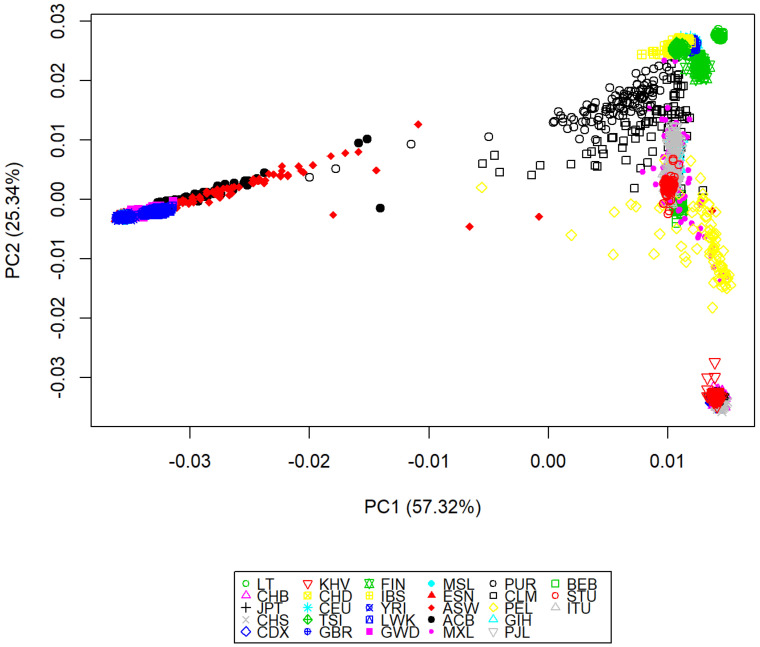
Principal component analysis of the first two PCs of individuals from Lithuania and 27 populations from the 1000 Genomes Project Phase3 dataset. Abbreviations as indicated in the text.

The first two PCs explained 57.32% and 25.34% of the variance, respectively. The results show all populations clustered according their continental origin (Figure 2).

### 3.3. Structural Variation

Summary statistics for structural variation (>49 bp) in parent and newborn groups are presented in Table 2. There was no statistically significant difference among parents and newborns in the analyzed structural variant groups. The average number of SVs was 9133 (not including newborns, as they inherit most of their variation from parents). On average there were 4159 (92% novel) deletions, 349 (93% novel) duplications, and 4621 (99% novel) insertions. Deletions and insertions are more abundant if compared with the number of duplications. 

### 3.4. De Novo Mutation Discovery

We further performed de novo variant (DNV) analysis. An exceptionally high number of de novo variants were identified for two trios (no. 2 and 4): 21,127 and 44,641, respectively. Their data were evaluated as an exclusion, thus, data for these trios were excluded from further analysis. In the final set of 23 trios, on average 158 single nucleotide variants and 34 indels (<49 bp) were identified. All participants had at least few de novo variants. Two de novo single nucleotides and two de novo indels were placed in chromosomes X and Y.

**Figure 3:**
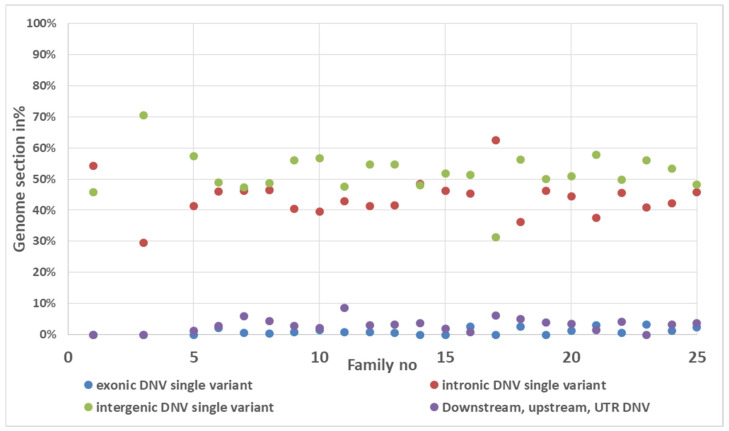
The distribution of de novo indels in genome regions according genome sequence function.

Analysis of 4417 DNVs identified by VarScan software showed that on average 1.1% de novo SNVs were exonic (37), 43.9% intronic (69), 51.9% intergenic (83), and the rest 3.13% in UTR or downstream sequence. Analysis of de novo indels revealed 1.07% exonic, 42.7% intronic, 48.4% intergenic, and 2.4% variants in UTR or downstream sequence (Figure 3). 

To assess whether there is a potentially pathogenic variant among the identified DNVs, predicted categorical scores for the damage induced by DNVs were analyzed. The following 10 values were considered: polyphen HDIV and HVAR, LRT, PROVEAN, CADD, FATHMM, MutationTester, MutationAssessor, SIFT, Fathmm-MKL coding, and GERP++. According to evaluated pathogenicity scores, three DNVs scored five or more estimates as pathogenic or potentially pathogenic therefore were identified them as possibly pathogenic: *ZSWIM8* (NM_001242487:c.3814G>C; p.G1272R), *CDC42EP1* (NM_152243:c.763C>A; p.P255T; rs77417880), and *RELA* (NM_001243985:c.1265A>G; p.N422S, rs746519095). All three DNVs are in a homozygous state.

## 4. Discussion

To study human genetics for many purposes, researchers intended to create a fully mapped sequence of the human genome and initiated the Human Genome Project (HGP) in 1990 [46]. Since then, the human reference genome has provided the foundation for genetic discovery and research, but recently, multiple authors of papers, such as Kaye A.M. et al., 2021 [47], Ballouz S. et al., 2019 [48], and Yang X. et al., 2019 [49], have highlighted the deficiencies of the linear reference, leading to a growing consensus that a richer reference structure is needed [47,48,49]. Continued improvements in the era of widespread whole-genome sequencing must improve the ability to predict how an individual’s inherited genome contributes to aging, complex disease, and even some monogenic diseases [50]. Furthermore, de novo mutations have increasingly been proposed to affect disease onset and progression [45]. As we move towards population-specific sequencing studies, the reference genome is no longer sufficiently static, with personalized reference genomes providing more accurate analysis results [51].

Here, in this article, we summarized the high-coverage WGS data obtained for this study that is analyzed and reported for the Lithuanian population for the first time. The data is of high quality with 36X coverage on average across the entire read length of 2 × 150 bp, with coverage distribution almost identical across all samples. WGS produced up to 757.973 M effective reads per sample (approximately 121 GB data per single sample run) (see Figure S2). A duplication rate of 5.21% in our WGS data is twice lower than in previously reported 10.12% Glanzmann et al. data [52], which indicates high levels of coverage for a target sequence, whereas high duplication rate indicates an enrichment bias. 

Primary analysis of the all variant allele frequency showed that the Lithuanian population distinguishes and is unique by alleles whose frequency in the gnomAd database is identified as rare or unique. The comparison results of the Lithuanian genome variants with Ashkenazi Jewish (AJN) ancestry genomes revealed that about half of the common variants with frequency 0.05–0.5 present in AJN genomes, in Lithuanian’s genomes become rare allelic variants with MAF < 0.05 (Figure S8). PCA analysis identified that Lithuanians position within the European context.

Novel SNVs and indel variants in 75 individuals were defined in the group of newborns and parents as absent from dbSNP build 150, respectively, 11.2% and 13.7%. Our study detected 4417 novel variants (SNs and indels) for 23 individuals, demonstrating the genetic diversity present in Lithuanian individuals. This finding underscores the value of sequencing Lithuanian individuals, as it allows the comprehensive cataloging and characterization of variants, which will in the future aid the clinical interpretation of genetic results [52]. 

In addition, during the study, we analyzed de novo variants. Aware of the fact that all individuals of the general population have only potentially neutral variants because survey subjects assessed themselves as “healthy” (although they may become ill in the future), we identified three potential pathogenic de novo variants in the *ZSWIM8*, *CDC42EP1*, and *RELA* genes for three different families. According to protein function prediction, *ZSWIM8* codes Zinc Finger SWIM-Type containing protein. Although we know that no pathological phenotype is expressed in the examined individual, disease such as Acromelic Frontonasal Dysostosis is associated with *ZSWIM8*. An important paralog of this gene is *ZSWIM6*. The frequency of identified NM_001242487: c.3814G>C rare allele was not previously reported. *CDC42EP1* codes for serum protein MSE55, which is a non-kinase CRIB (Cdc42/Rac interactive-binding) domain-containing molecule of unknown function. These findings indicate that MSE55 is a Cdc42 effector protein that mediates actin cytoskeleton reorganization at the plasma membrane. The third DNV was in the *RELA* gene, which encodes NF-kappa-B, a ubiquitous transcription factor involved in several biological processes. It is held in the cytoplasm in an inactive state by specific inhibitors. Upon degradation of the inhibitor, NF-kappa-B moves to the nucleus and activates transcription of specific genes. NF-kappa-B is composed of NFKB1 or NFKB2 bound to either REL, RELA, or RELB. The most abundant form of NF-kappa-B is NFKB1 in a complex with the product of this gene, RELA. Four transcript variants encoding different isoforms have been found for this gene. Despite the pathogenicity prediction the persistence of the variant in the genome and the absence of effect phenotypes are due to many factors, therefore, it is necessary to examine the variant in appropriate conditions and of course to confirm by Sanger sequencing. Those include environment, age of parents, genomic context, epigenetics, and other factors because all of them influence the value of mean relative fitness that increases monotonically, whereas the strength of selection decreases [53].

In summary, WGS follows the current trends in the convergence of fundamental genomic research and clinical implications of the presence or absence of certain genes. Now, WGS is becoming one of the most widely used applications and is providing tremendous quantities of genome sequences relative to the past through public and private human genome sequencing projects throughout the world. The ability to compare and contrast genomic findings across a unified database containing information from diverse populations is crucial to advancing our understanding of the human genome [47]. 

## Figures and Tables

**Table 1:** The average number of autosomal single nucleotide genetic variants per genome identified in the Lithuanian cohort.

	Parents (*n* = 50)	Newborns (*n* = 25)
Raw reads in M	78,447	83,755
Bases (Gb)	11,832	12,633
Coverage depth	35.46	37.88
SNVs	4,704,096	4,696,226
SNPs	3,791,674	3,783,578
Insertions (Hom)	155,784	153,755
Insertions (Het)	277,288	276,262
Deletions (Hom)	152,914	150,766
Deletions (Het)	293,193	291,467
Indels (Het)	22,332	22,377

**Table 2:** Summary statistics for deletions, duplications, and insertions in parent and newborn groups.

	Parents (*n* = 49)	Newborns (*n* = 24)	
Statistics	Deletions		*p*-Value
Mean	4159.39	4191.33	0.5611
SE	33.63	47.08	
Median	4126.0	4172.5	
Mode	3963	#N/A	
SD	23,543	23,064	
	**Duplications**		
Mean	34,884	35,408	0.2747
SE	3.94	4.76	
Median	346	356	
Mode	365	355	
SD	27.61	23.31	
	**Insertions**		
Mean	4620.49	4661.54	0.5033
SE	49.98	63.05	
Median	4612.0	4697.5	
Mode	#N/A	#N/A	
SD	34,983	30,889	

SE—standard error, SD—standard deviation.

## Data Availability

The WGS variation data have been deposited on https://figshare.com/articles/dataset/Inherited_and_de_novo_variation_in_Lithuanian_genomes_introduction_to_the_analysis_of_the_generational_shift/19354817 (accessed on 14 March 2022).

## Supplementary Materials

The following supporting information can be downloaded at: https://www.mdpi.com/article/10.3390/genes13040569/s1, Figure S1: Sequence lengths of trimmed FASTQ reads (average of all samples) (DNA); Figure S2: Sequence quality of trimmed FASTQ reads (average of all samples) (DNA); Figure S3: GC content of trimmed FASTQ reads (average of all samples) (DNA); Figure S4. Principal component analysis (PCA) of 50 individuals (parents) from Lithuania included in the study. Figure S5. Principal component analysis (PCA) of 25 newborns from Lithuania included in the study. Figure S6. Summary of the identified variants. Distribution of SNPs and indels. Figure S7. Summary of the identified variants. Distribution of structural variants. Figure S8. Allele frequency distribution comparison between the Lithuanian and African American, Latino ancestry, Finish, uncertain, Ashkenazi Jewish, and east Asian ancestry genomes. Table S1: Statistics of mapped reads (DNA); Tables S2–S4: Number of identified variants (passing QC filter); Table S5: Relatedness analysis performed for trios; Table S6: Detected outliers in our study.
