# Supplementary material for: Inherited and De Novo Variation in Lithuanian Genomes: Introduction to the Analysis of the Generational Shift
Source: Genes (Basel). 2022 Mar 23;13(4):569. doi: 10.3390/genes13040569 (PMC9028680; doi:10.3390/genes13040569)
Supplement: Supplementary file 1 [file genes-13-00569-s001.zip › Suppl Figures-genes.pdf]

Supplementary Material

Inherited and de novo variation in Lithuanian genomes: introduction to the analysis of the generational shift

Alina Urnikyte[1*], Laura Pranckeniene[1*], Ingrida Domarkiene[1], Svetlana Dauengauer-Kirliene[1], Alma Molyte[1], Ausra Matuleviciene[1], Ingrida Pilypiene[1], Vaidutis Kučinskas[1]

[1]Department of Human and Medical Genetics, Biomedical Science Institute, Faculty of Medicine, Vilnius University, Santariskiu Street 2, LT-08661, Vilnius, Lithuania; alina.urnikyte@mf.vu.lt; laura.pranckeniene@mf.vu.lt; ingrida.domarkiene@mf.vu.lt; svetlana.dauengauer-kirliene@mf.vu.lt; alma.molyte@mf.vu.lt; ausra.matuleviciene@mf.vu.lt; ingrida.pilypiene@mf.vu.lt; vaidutis.kucinskas@mf.vu.lt
*Correspondence: alina.urnikyte@mf.vu.lt; laura.pranckeniene@mf.vu.lt

Supplementary Figures

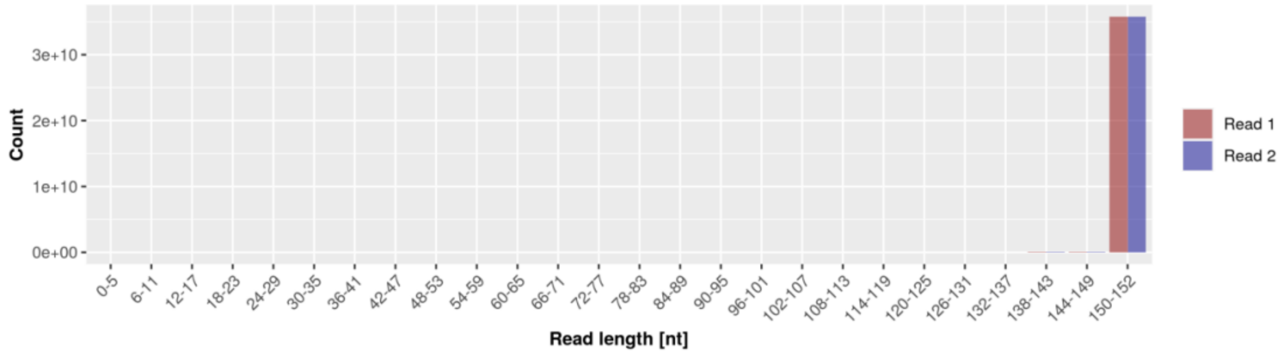

Figure S1. Sequence lengths of trimmed FASTQ reads (average of all samples) (DNA).

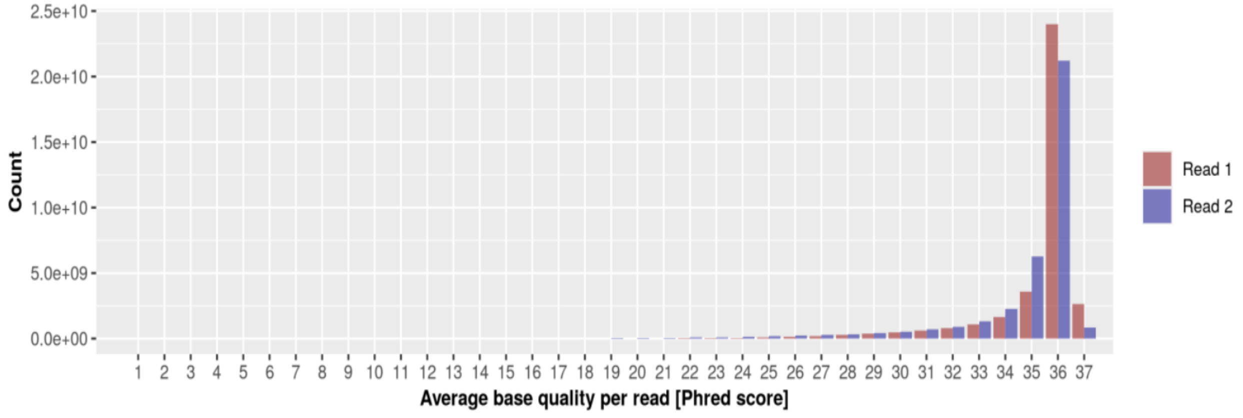

Figure S2. Sequence quality of trimmed FASTQ reads (average of all samples) (DNA).

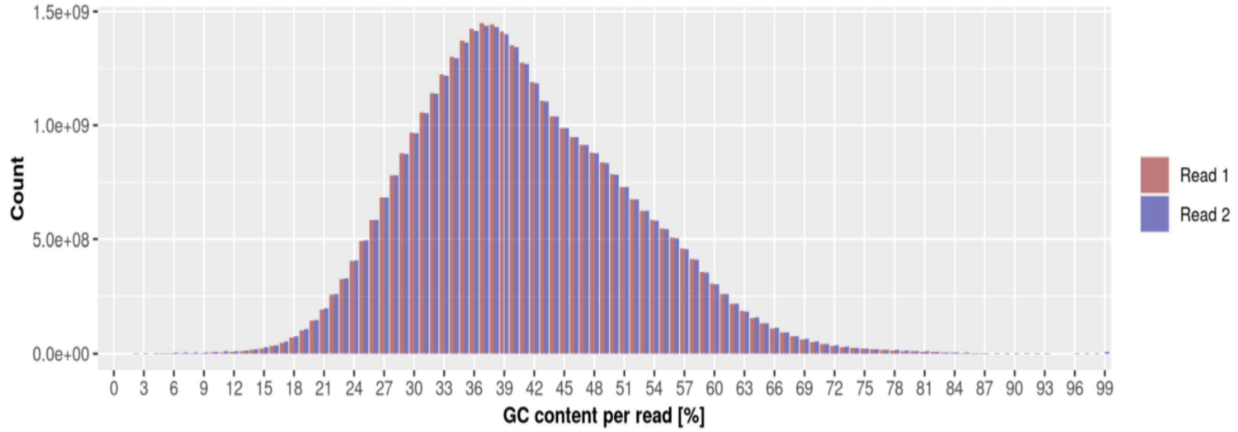

Figure S3. GC content of trimmed FASTQ reads (average of all samples) (DNA).

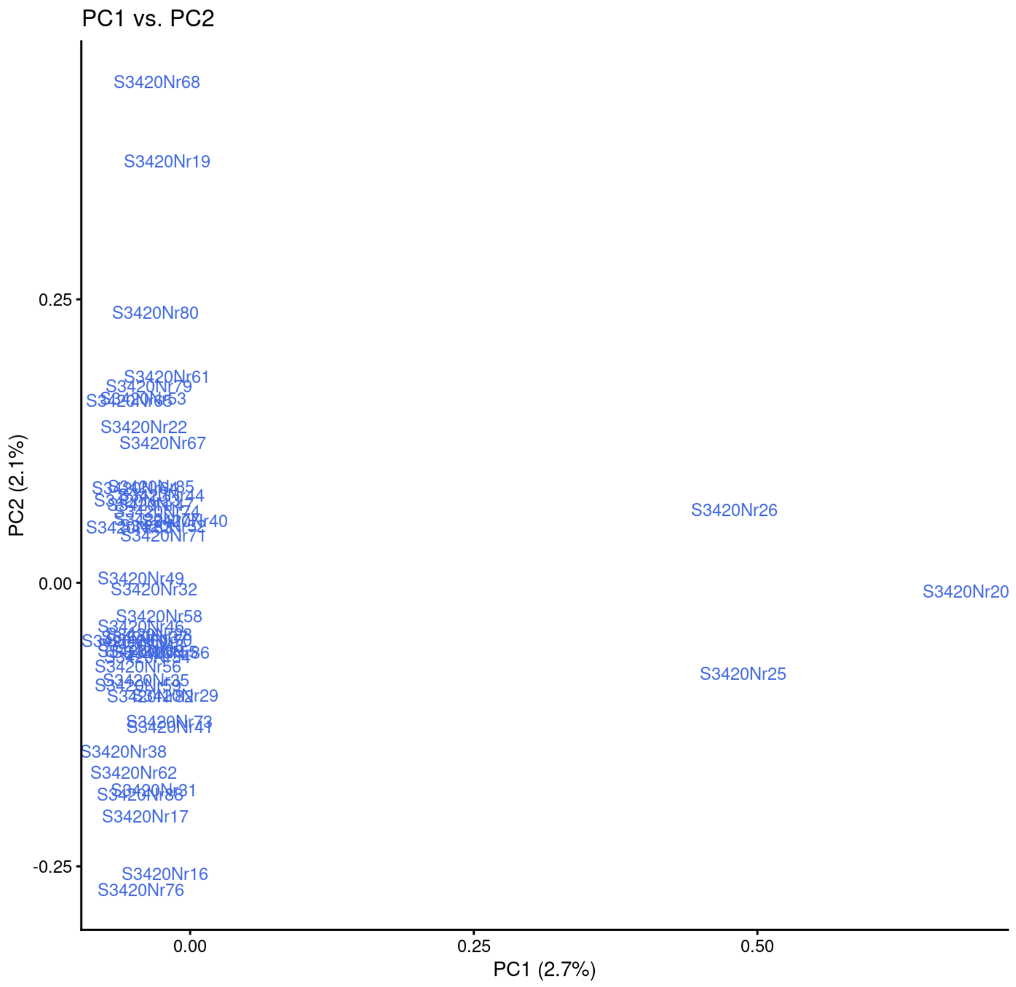

Figure

Figure S4. Principal component analysis (PCA) of 50 individuals (parents) from Lithuania included in the study.

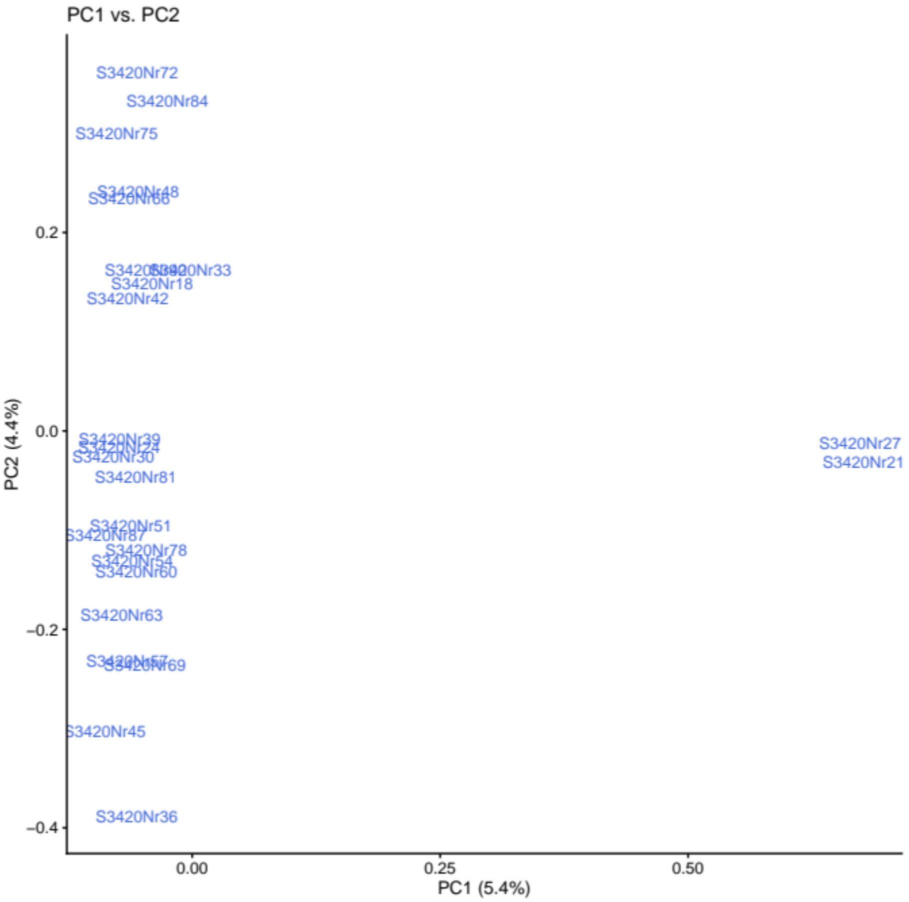

Figure S5. Principal component analysis (PCA) of 25 newborns from Lithuania included in the study.

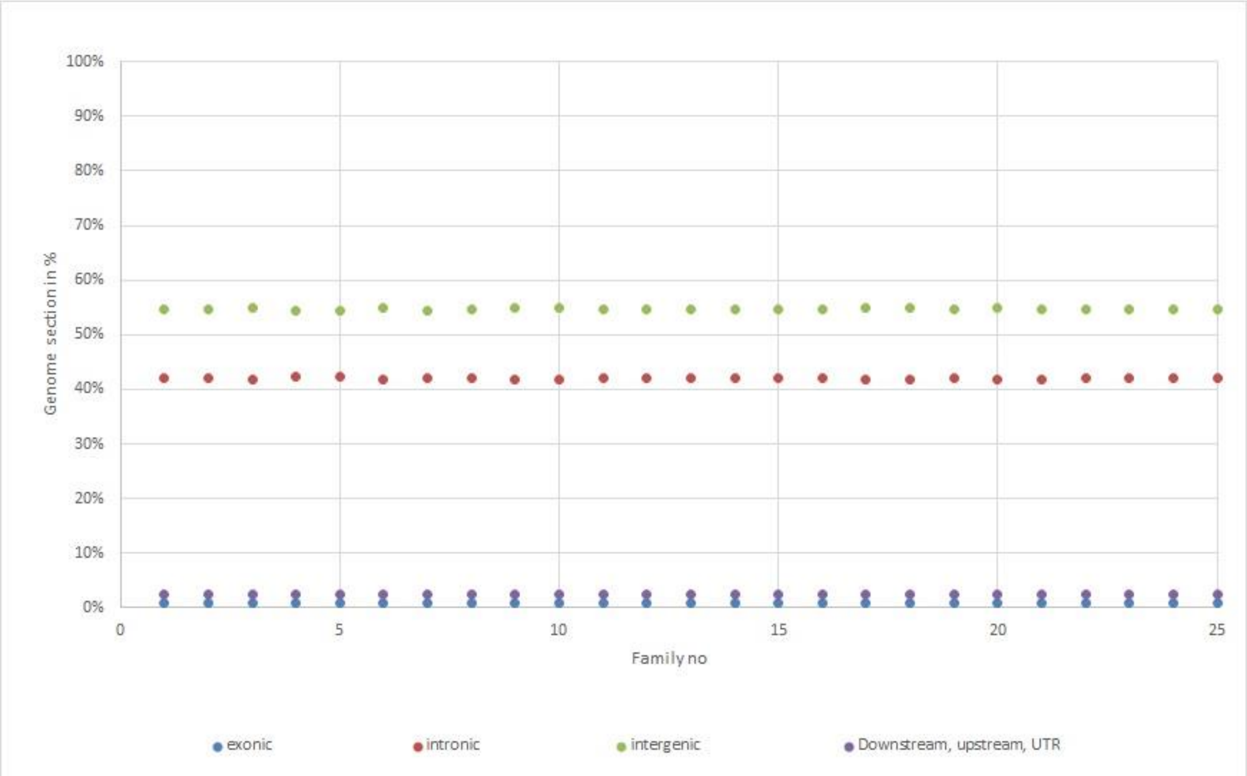

Figure S6. Summary of the identified variants. Distribution of SNPs and indels.

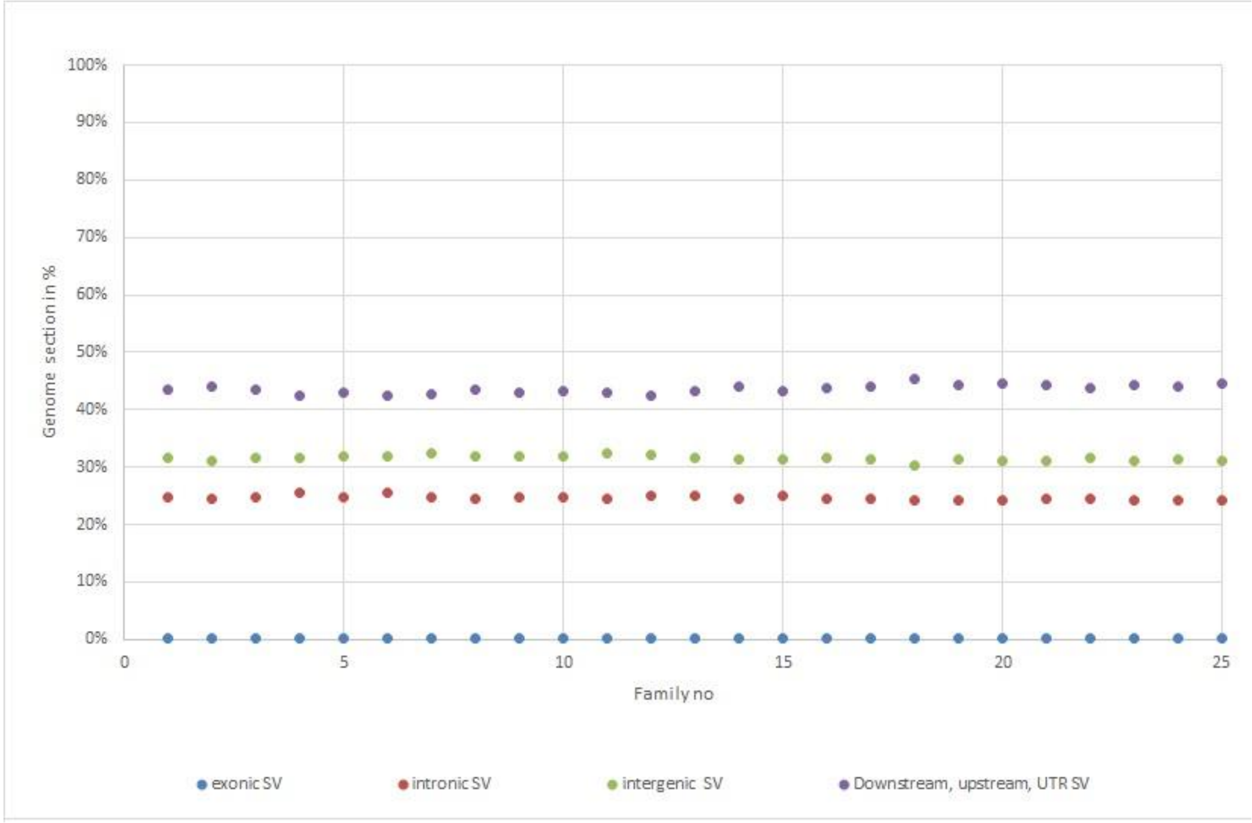

Figure S7. Summary of the identified variants. Distribution of structural variants.

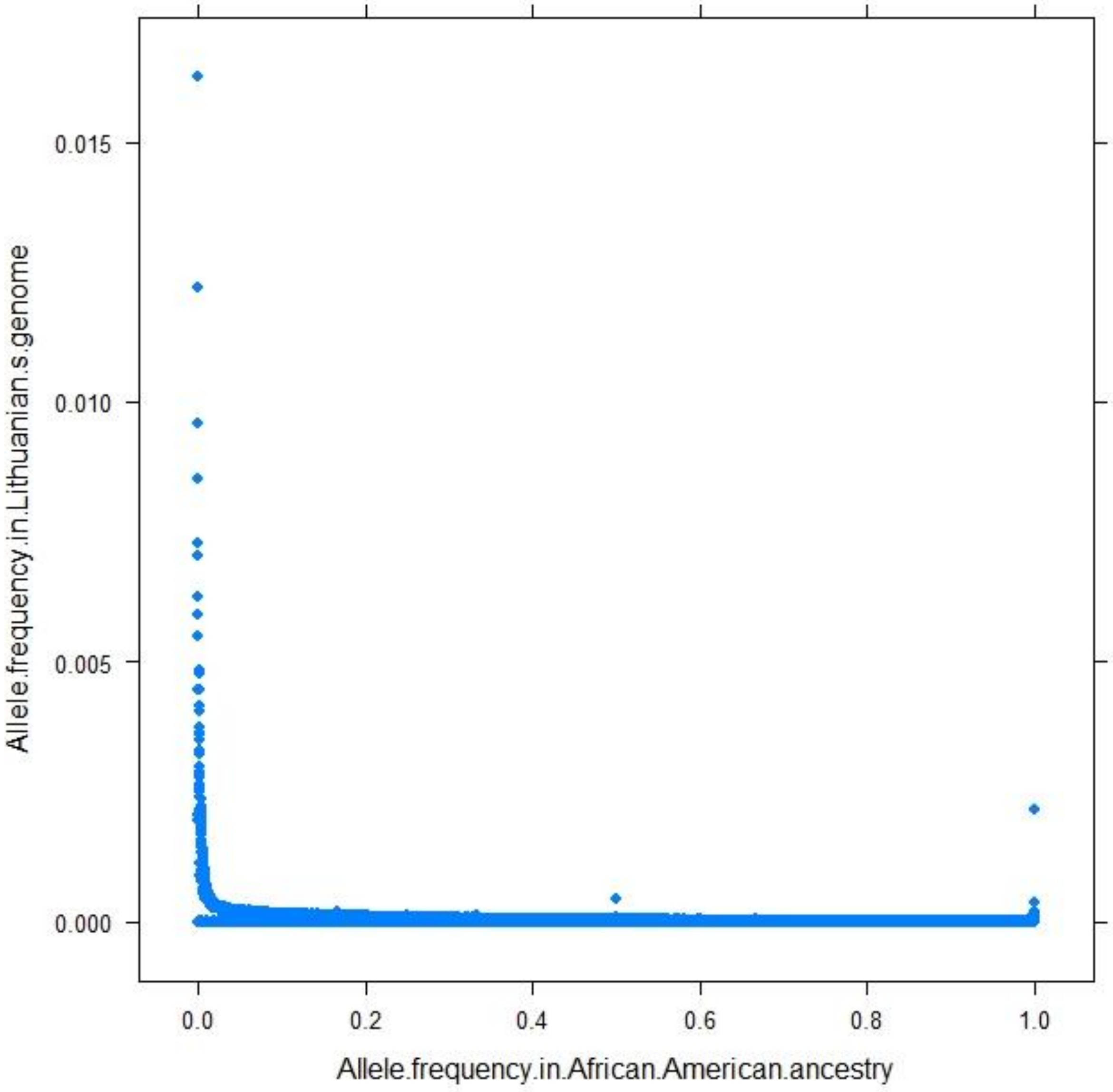

0.015
0.010
0.005
0.000
Allele.frequency.in.Lithuanian.s.genome
0.0
0.2
0.4
0.6
0.8
1.0
Allele.frequency.in.African.American.ancestry

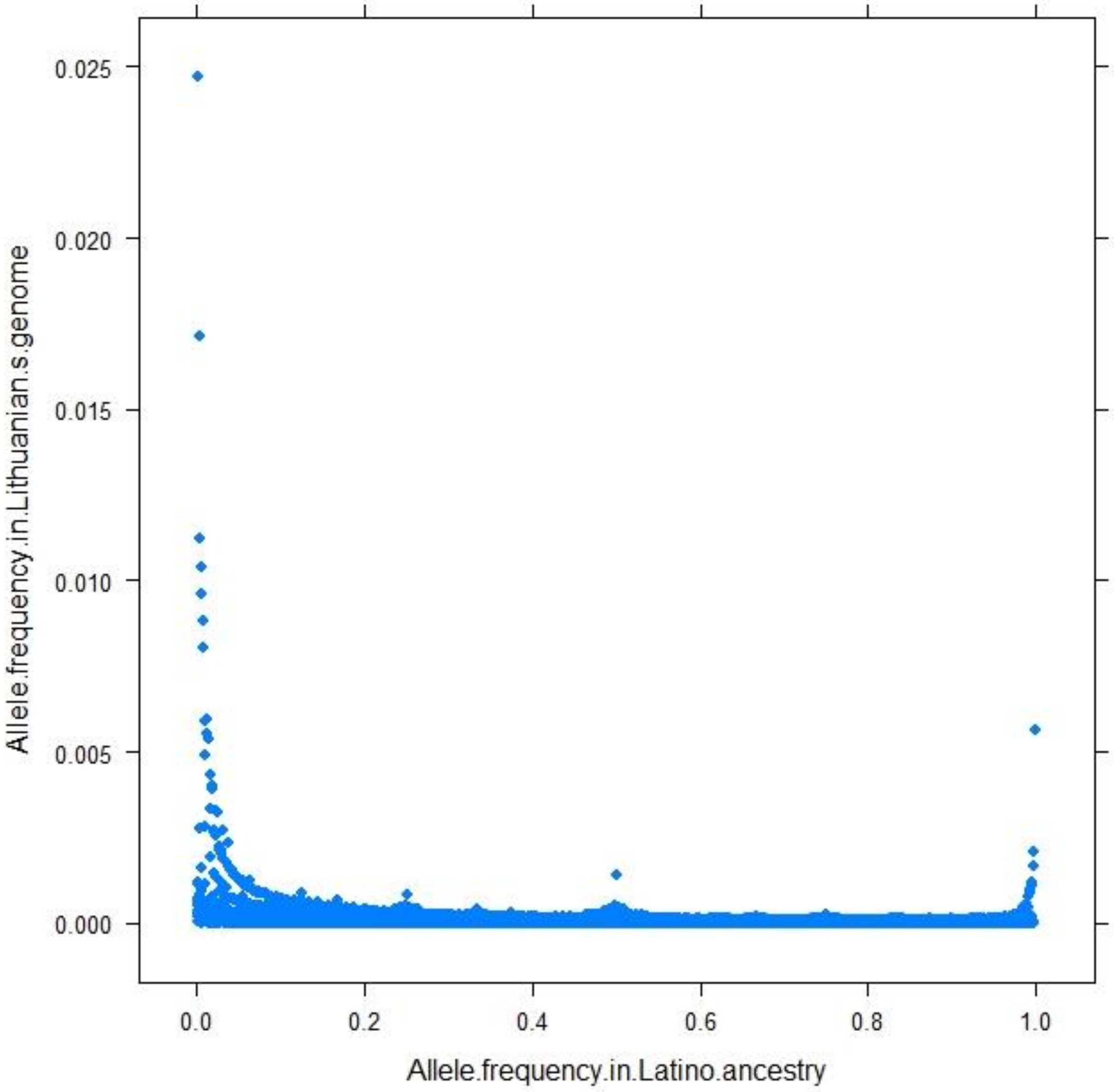
0.025
0.020
0.015
0.010
0.005
0.000
0.0
0.2
0.4
0.6
0.8
1.0
Allele.frequency.in.Lithuanian.s.genome
Allele.frequency.in.Latino.ancestry

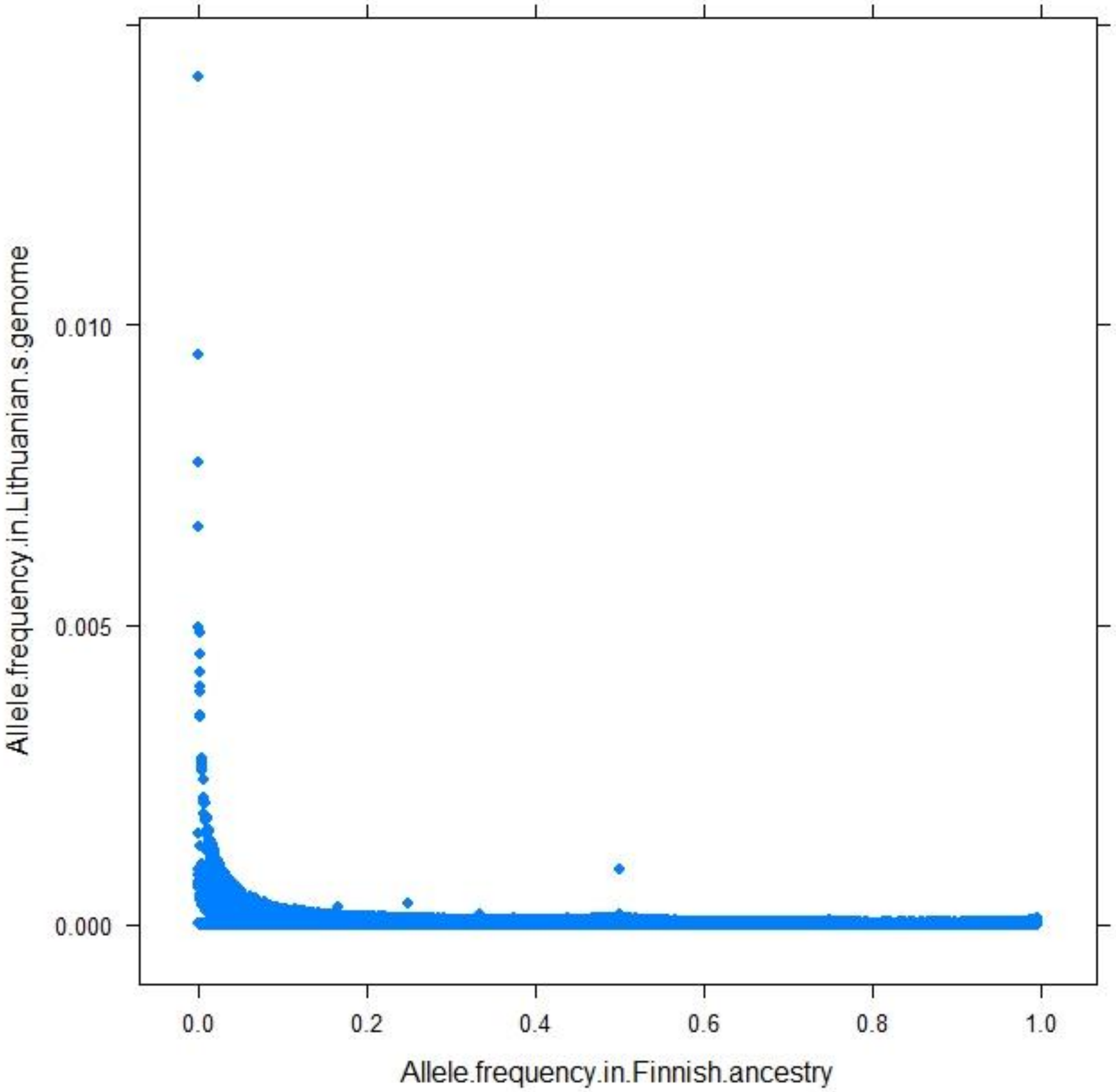

Allele.frequency.in.Lithuanian.s.genome
0.010
0.005
0.000
0.0
0.2
0.4
0.6
0.8
1.0
Allele.frequency.in.Finnish.ancestry

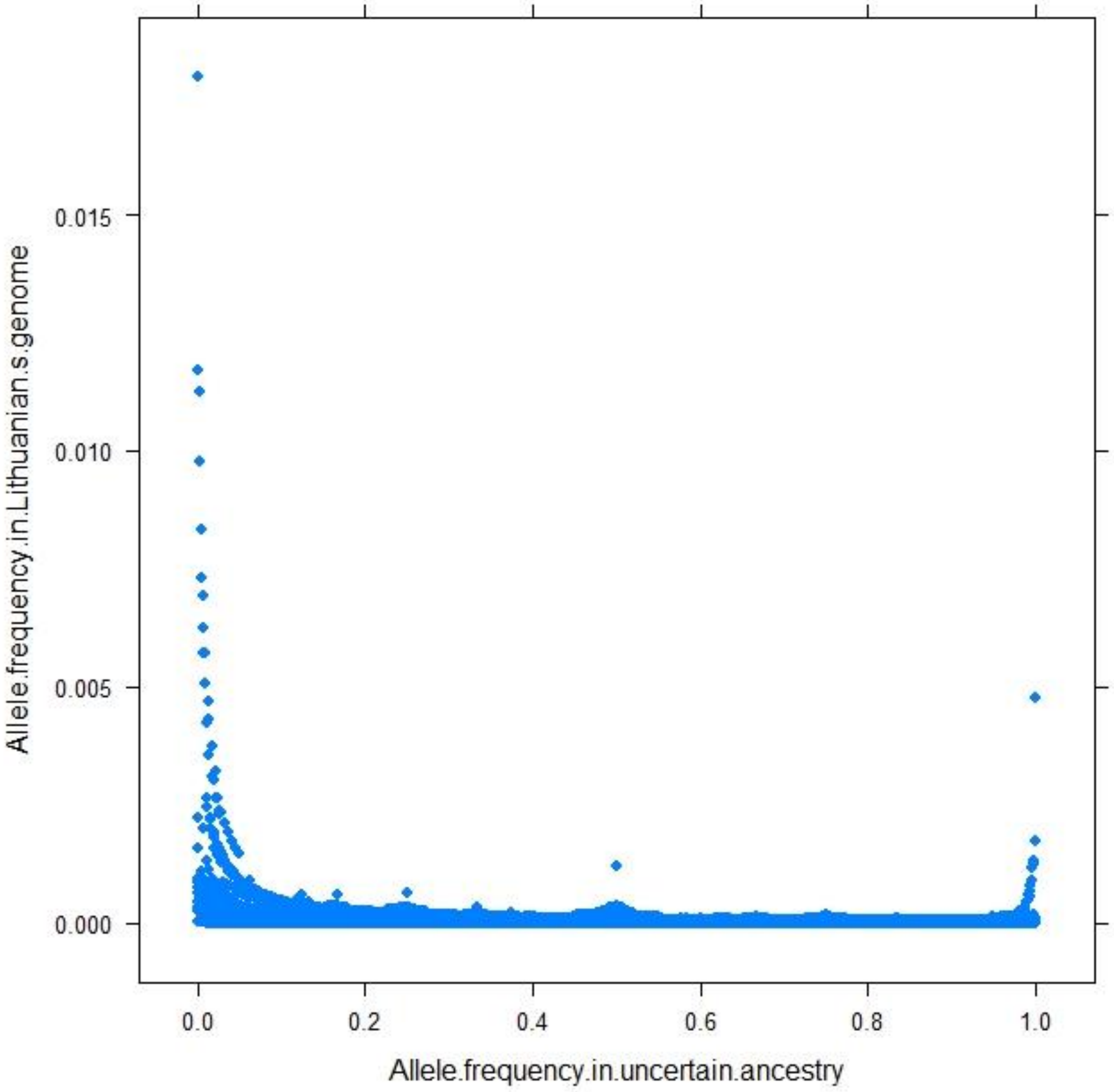

0.015
0.010
0.005
0.000
Allele.frequency.in.Lithuanian.s.genome
0.0
0.2
0.4
0.6
0.8
1.0
Allele.frequency.in.uncertain.ancestry

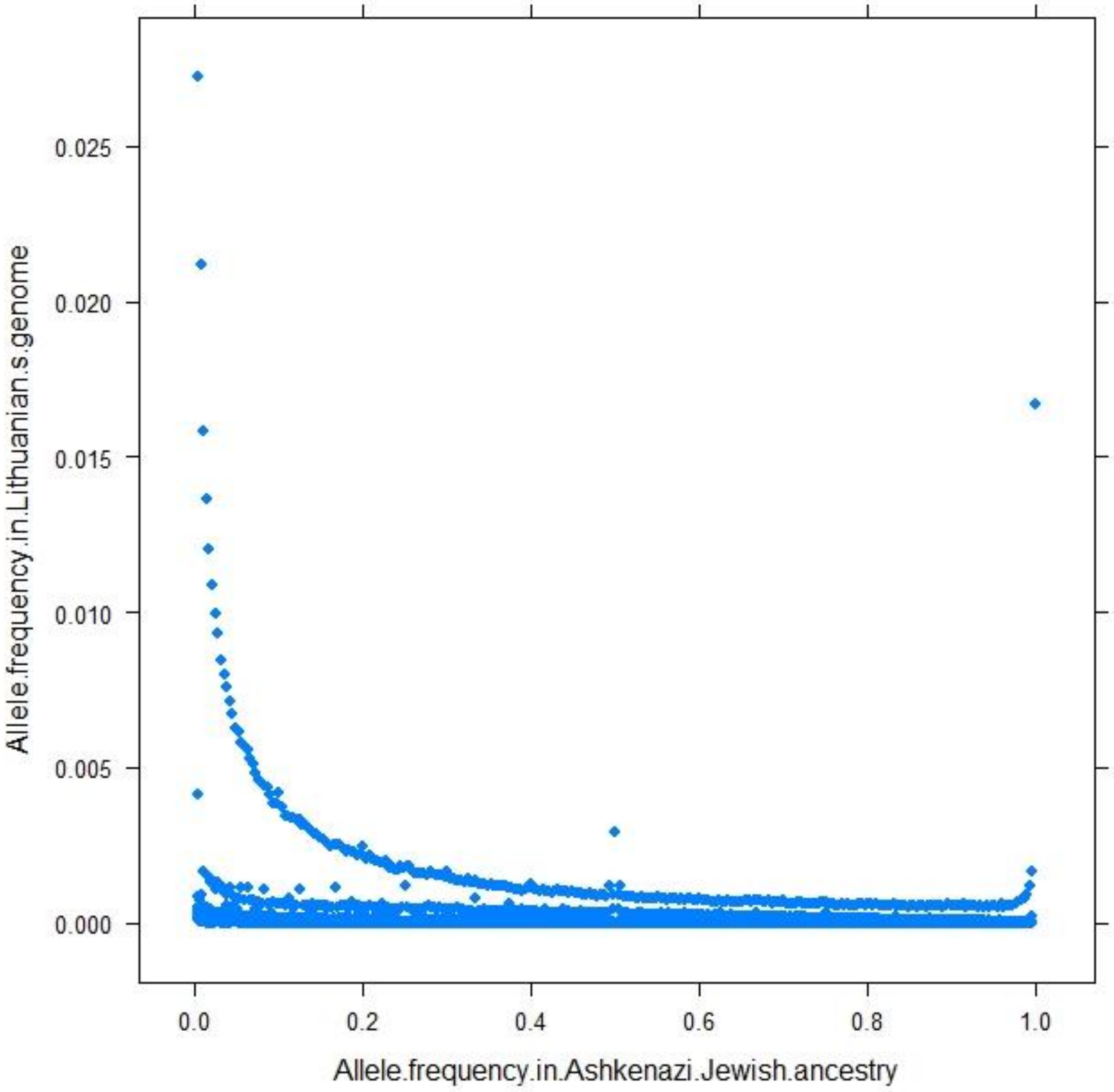

0.025
0.020
0.015
0.010
0.005
0.000
Allele.frequency.in.Lithuanian.s.genome
0.0
0.2
0.4
0.6
0.8
1.0
Allele.frequency.in.Ashkenazi.Jewish.ancestry

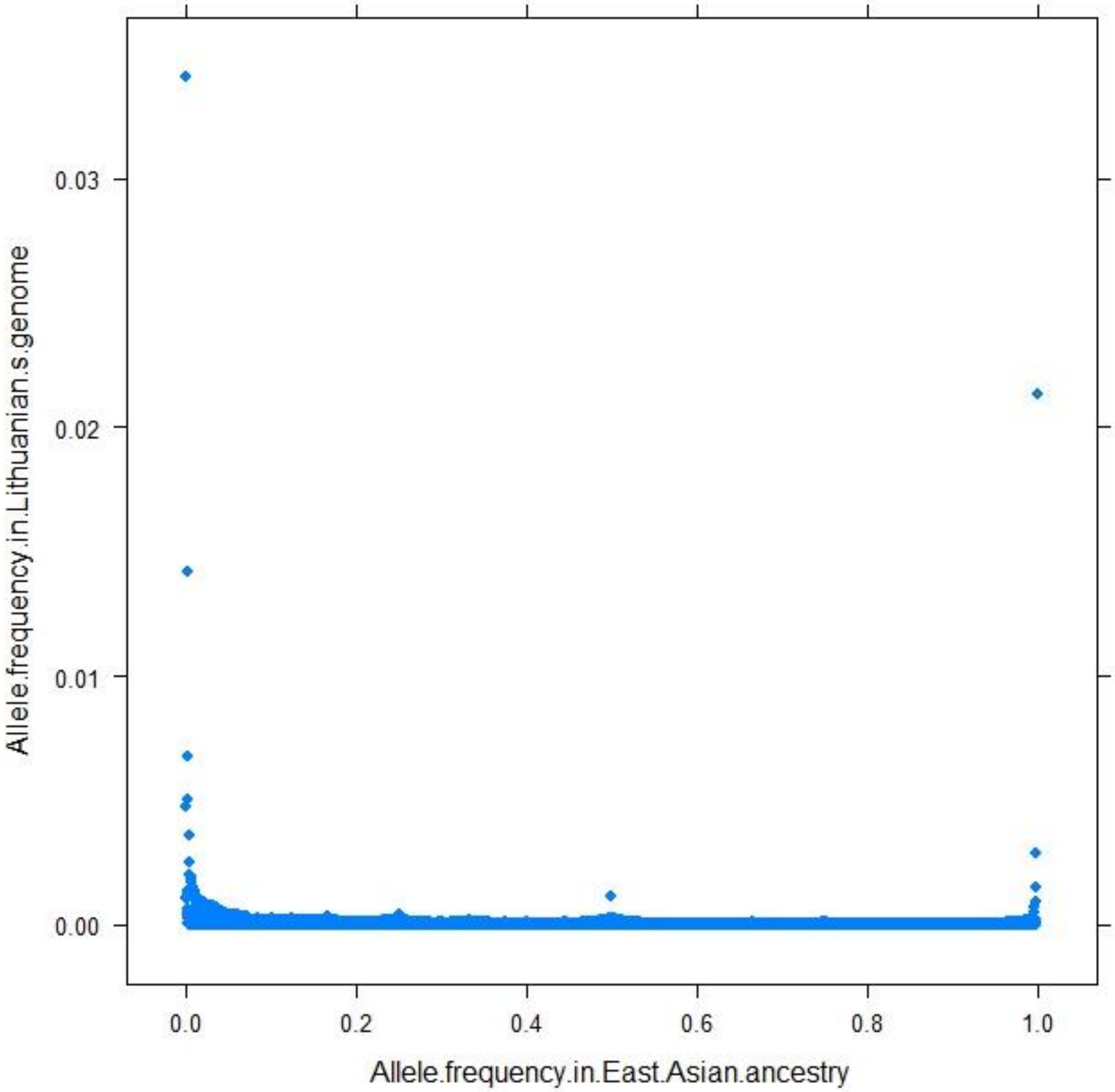
0.03
0.02
0.01
0.00
Allele.frequency.in.Lithuanian.s.genome
0.0
0.2
0.4
0.6
0.8
1.0
Allele.frequency.in.East.Asian.ancestry

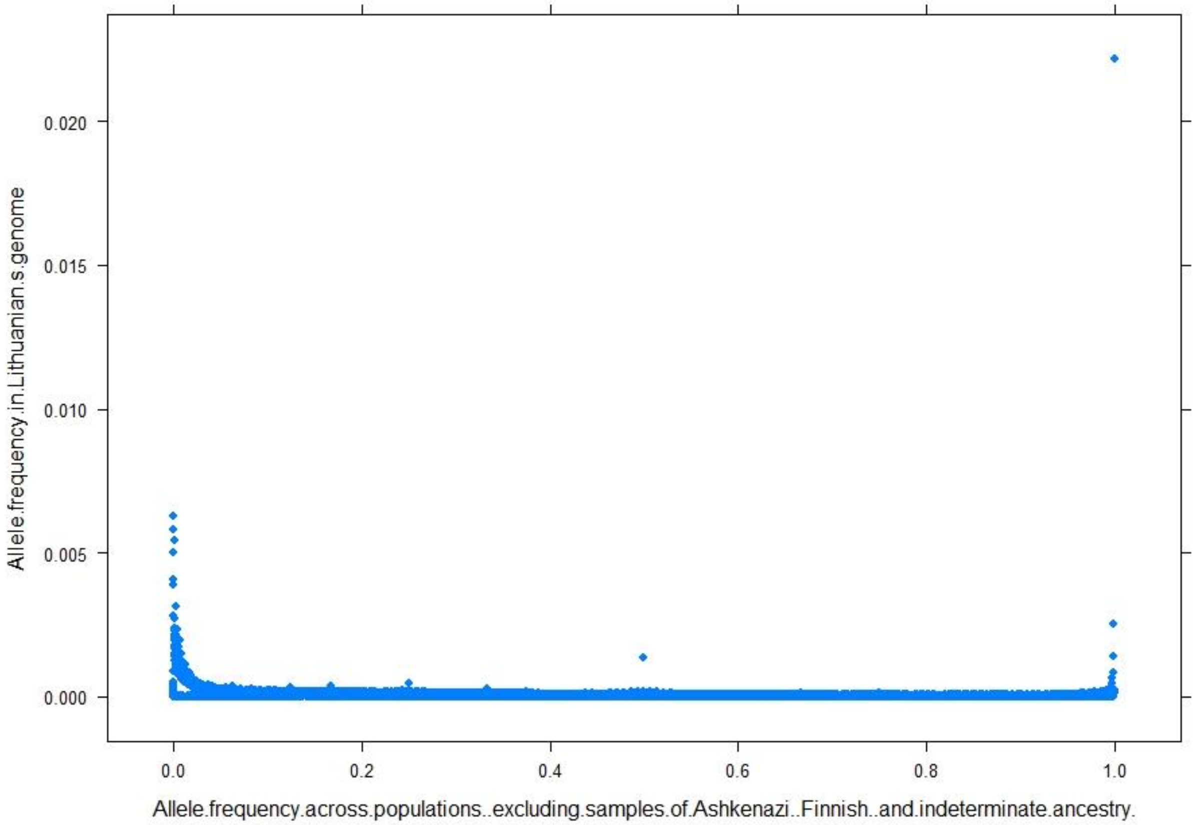

Figure S8. Allele frequency distribution comparison between the Lithuanian and African-American, Latino ancestry, Finish, uncertain, Ashkenazi Jewish, and east Asian ancestry genomes.
